# The Effects of Different Degrees of Procyanidin Polymerization on the Nutrient Absorption and Digestive Enzyme Activity in Mice

**DOI:** 10.3390/molecules23112916

**Published:** 2018-11-08

**Authors:** Huairong Zhong, Yong Xue, Xiaoyuan Lu, Qiang Shao, Yuelei Cao, Zhaoxia Wu, Gao Chen

**Affiliations:** 1 Biotechnology Research Center, Shandong Academy of Agricultural Sciences, Ji’nan 250100, China; zh.hr12@163.com (H.Z.); luxiaoyuan1225@163.com (X.L.); shaoqiang12345@126.com (Q.S.); caoyuelei0733@163.com (Y.C.); 2 Shandong Provincial Key Laboratory of Genetic Improvement, Ecology and Physiology of Crops, Ji’nan 250100, China; 3 Department of Synthetic Biology and Bioenergy, J. Craig Venter Institute, Rockville, MD 20850, USA; yongxue@gmail.com; 4 College of Food Science, Shenyang Agricultural University, Shenyang 110866, China

**Keywords:** antioxidant properties, digestive enzyme, nutrient, polymerization, proanthocyanidins

## Abstract

Proanthocyanidins, including polymers with both low and high degrees of polymerization, are the focus of intensive research worldwide due to their high antioxidant activity, medicinal applications, and pharmacological properties. However, the nutritional value of these compounds is limited because they readily form complexes with proteins, polysaccharides, and metal ions when consumed. In this study, we examined the effects of proanthocyanidins with different degrees of polymerization on white mice. Twenty-four male white mice were randomly divided into three groups of eight mice each and fed proanthocyanidins with a low degree of polymerization or a high degree of polymerization or a distilled water control via oral gavage over a 56-day period. We examined the effects of these proanthocyanidins on digestive enzyme activity and nutrient absorption. Compared to the control group, the group fed high-polymer proanthocyanidins exhibited a significant reduction in net body mass, total food intake, food utility rate, amylase activity, protease activity, and major nutrient digestibility (*p* < 0.05), while the group fed low-polymerization proanthocyanidins only exhibited significant reductions in total food intake, α-amylase activity, and apparent digestibility of calcium and zinc (*p* < 0.05). Therefore, proanthocyanidins with a high degree of polymerization had a greater effect on digestive enzyme activity and nutrient absorption than did those with a low degree of polymerization. This study lays the foundation for elucidating the relationship between procyanidin polymerization and nutrient uptake, with the aim of reducing or eliminating the antinutritional effects of polyphenols.

## 1. Introduction

Proanthocyanidins are a large class of polyphenolic compounds produced by a wide range of plants. These compounds are composed of a mixture of molecules with different degrees of polymerization, including catechins, epicatechins, and epicatechin gallates linked by C4-C6 or C4-C8 bonds. Polymers composed of two to four monomer units are classified as oligomers while those composed of five or more monomers are high polymers [1]. Proanthocyanidins are the most potent antioxidants and free radical scavengers identified to date; the antioxidant capacity of proanthocyanidins is 20-times that of vitamin C and 50-times that of vitamin E [2]. Most pharmacological functions of proanthocyanidins, such as their anti-cancer, anti-aging, and anti-inflammatory properties and their ability to lower blood pressure, blood fat levels, and blood sugar levels, are closely related to their strong antioxidant activity.

Numerous studies conducted worldwide have examined the physiological activity and medicinal value of proanthocyanidins [3,4,5], and these compounds have increasingly been used to prevent and treat disease. However, due to their polyhydroxy structure, proanthocyanidins readily undergo complexation reactions with proteins, polysaccharides, and metal ions, reducing the nutritional value of these compounds [6,7]. Polyphenols are widely distributed in nature and are present in plant-derived foods, teas, medicines, and animal feed. However, when polyphenols are consumed, they combine with other compounds, which reduces their nutritional value and limits their development as human health and animal husbandry products [8]. The molecular weight of polyphenols affects their ability to bind to proteins; polyphenols with a molecular weight of less than 500 kD rarely precipitate with proteins in vitro [9].

Several recent studies have examined the effects of the degree of proanthocyanidin polymerization on food functional properties, such as foaming and foam stability. Proanthocyanidins with a higher degree of polymerization have a stabilizing effect on the foaming properties of proteins and are better able to bind to proteins than are those with a low degree of polymerization. The polyhydroxyl properties of proanthocyanidins enable them to bind to multiple protein sites simultaneously [10]. A study of apple juice turbidity revealed that juice proteins aggregated with catechin polymers but not with catechin or epicatechin monomers, suggesting that low-molecular-weight proanthocyanidins do not effectively crosslink proteins [11].

To date, most studies examining the effects of polyphenols with different molecular weights on the digestion and absorption of nutrients have been performed in vitro. Stojadinovic et al. [12] simulated the gastrointestinal digestive environment to study the mechanism by which polyphenols and β-proteins interact, and found that non-covalent bonding between polyphenols and proteins inhibits the digestion of β-lactoglobulin by pepsin and trypsin; the higher the binding strength, the slower the rate of protein digestion. Gonçalves et al. [13] confirmed that t high-polymer proanthocyanidins inhibit α-amylase activity to a greater extent than do oligomeric proanthocyanidins in vitro, due to the broader interaction between α-amylase and homomeric proanthocyanidins. Furthermore, Baxter et al. [14] showed that insoluble aggregates resulting from an interaction between polyphenols and proline-rich proteins contributed to sputum formation. Sarni-Manchadoy et al. [15] confirmed that the precipitate mainly forms in vitro via the interaction between high-polymer polyphenols and salivary proteins, perhaps because the higher hydroxyl content of high-polymer polyphenols enables them to interact with salivary proteins more extensively than with low-polymer polyphenols.

Although the results of in vitro simulation experiments are somewhat correlated with the results of in vivo tests, these experiments do not fully account for the actual digestion and absorption processes of the body. Therefore, in the current study, we explored the relationship between the degree of procyanidin polymerization and nutrient uptake in mice. The results of this study lay the foundation for reducing or eliminating the antinutritional effects of polyphenols.

## 2. Results

### 2.1. Effects of Proanthocyanidins with Different Degrees of Polymerization on Body Weight in Mice

The initial body weights of mice in the experimental groups were not significantly different from those of the control group, However, after a 56-day treatment with high- or low-polymer proanthocyanidins or a distilled water control, the body weight of mice in the high-polymer group was significantly lower than that of the control group (*p* < 0.05), whereas that of mice in the low-polymer group was not (*p* > 0.05; Table 1). These results indicate that proanthocyanidins with a high degree of polymerization affect the normal growth and development of mice.

The total food intake of the high-polymer group was significantly lower than that of the control group (*p* < 0.05), indicating that the proanthocyanidin solution inhibited food intake by the mice. Perhaps the activity of digestive enzymes secreted by the mice and of enzymes produced by microorganisms in their digestive tracts was reduced through their interaction with the polyphenols, and this affected satiety and levels of fiber and insoluble compounds, resulting in a reduction in food intake [16]. 

We calculated the net mass gain of the mice based on their initial and final body masses and the food utility rate of the mice using formula (1) (Table 1). The food utility rate was significantly lower for mice in the high-polymer group vs. the control group (*p* < 0.05). Although the food utility rate of mice in the low-polymer group was lower than that of the control group, the difference was not significant (*p* > 0.05). These results indicate that high-polymer proanthocyanidins reduce the food utilization rate of mice. These results suggest that high-polymer proanthocyanidins have a stronger tendency than low-polymer proanthocyanidins to form complexes with enzymes and biological macromolecules such as proteins, fats, and sugars, resulting in a lower nutrient utilization rate.

### 2.2. Effects of Proanthocyanidins with Different Degrees of Polymerization on Digestive Enzyme Activity

We calculated the inhibitory effects of proanthocyanidins with different degrees of polymerization on digestive enzyme activity using Formula (2). The effects of these treatments on the activities of various digestive enzymes are described below.

#### 2.2.1. α-Amylase Activity

The α-amylase activity in the small intestine and pancreas was significantly lower in the experimental groups than the control group (*p* < 0.05; Table 2). This enzyme activity was also significantly lower in mice in the high- vs. low-polymer group (*p* < 0.05). In the small intestine, α-amylase activity was inhibited at a rate of 21% in the low-polymer group and 41% in the high-polymer group. In the pancreas, α-amylase activity was inhibited at a rate of 26% in the low-polymer group and 45% in the high-polymer group. The α-amylase digestive enzyme activity was significantly lower in the experimental groups than in the control group, likely because the large number of phenolic hydroxyl groups in the polyphenols interacted with the peptidyl-NH-CO-, amino-NH_2_-, and carboxyl-COOH groups of the enzymes in the form of hydrogen bonds, forming a non-digestible complex that caused the catalytic activity of the enzymes to be reduced or lost. α-amylase activity was significantly lower in mice in the high- vs. low-polymer group, likely because the proanthocyanidins with high degrees of polymerization had more sites that bind with α-amylase, increasing the number of interactions between these molecules.

#### 2.2.2. Trypsin and Pepsin Activity

The trypsin and pepsin activity in the small intestine, pancreas, and stomach was significantly lower in the high-polymer group compared to the control (*p* < 0.05; Table 2). Although this activity was lower in the low-polymer group than in the control, the difference was not significant (*p* > 0.05). Perhaps proanthocyanidins with a high degree of polymerization were more susceptible to binding by proteases than were the other proanthocyanidins. In the small intestine, trypsin activity was inhibited at a rate of 15% in the low-polymer group and 32% in the high-polymer group. In the pancreas, trypsin activity was inhibited at a rate of 21% in the low-polymer group and 39% in the high-polymer group. In the stomach, pepsin activity was inhibited at a rate of 13% in the low-polymer group and 38% in the high-polymer group.

#### 2.2.3. Pancreatic Lipase Activity

Pancreatic lipase activity in the small intestine and pancreas was lower in the experimental groups than in the control group, but the difference was not significant (*p* > 0.05; Table 2), suggesting that both groups of proanthocyanidins had a small inhibitory effect on lipase activity. In the small intestine, pancreatic lipase activity was inhibited at a rate of 7% in the low-polymer group and 10% in the high-polymer group. In the pancreas, pancreatic lipase activity was inhibited at a rate of 10% in the low-polymer group and 13% in the high-polymer group. Compared to amylase and protease activity, proanthocyanidins had a smaller effect on pancreatic lipase activity, perhaps because pancreatic lipase has a weaker affinity for these compounds than the other digestive enzymes.

### 2.3. Effects of Proanthocyanidins with Different Degrees of Polymerization on Nutrient Digestibility

Proanthocyanidins with different degrees of polymerization had different effects on the apparent digestibility of proteins. The apparent digestibility of proteins was significantly lower in the high-polymer group than in the control group (*p* < 0.05; Table 3), but there was no significant difference in this value between the low-polymer and control group (*p* > 0.05). The decrease in apparent protein digestibility in the high-polymer group might have been due to the binding of polyphenols to proteases in mice, resulting in the inhibition of enzyme activity, a reduction in protein digestion, and, ultimately, a decrease in protein digestibility in vivo. In addition, polyphenols can directly combine with macromolecular proteins to form molecular complexes that are not easily digested and absorbed by the human body, reducing the effective utilization of nitrogen by intestinal microbes and ultimately reducing the utilization of proteins in food. Proteins are also major components of the cell membrane. Polyphenols can reduce the permeability of the plant cell membrane by binding to macromolecular substances on the cell surface, reducing the solubility of nutrients in the cell and ultimately decreasing the digestibility of proteins in feed. The apparent digestibility of protein was significantly lower in the high- vs. low-polymer group, perhaps because polyphenols containing more hydroxyl groups or with a higher molecular weight react more readily with proteins. Although polyphenols bind to proteins, this binding is selective. The molecular mass, spatial configuration, and other aspects of polyphenols affect their reaction with proteins.

### 2.4. Effects of Proanthocyanidins with Different Degrees of Polymerization on Fat Digestibility

The apparent digestibility of fat was significantly lower in the high polymerization group than the control group (*p* < 0.05; Table 3), but there was no significant difference between these values in the low-polymer group vs. the control (*p* > 0.05). The decrease in lipid digestibility in the high-polymer group might have been due to the strong complexation of polyphenols with lipid macromolecules, which hindered the biodegradation of lipids and ultimately led to a decrease in fat digestibility. In addition, polyphenols could bind to phospholipids, proteins, and polysaccharides on the surfaces of cell membranes, thereby reducing the permeability of the membrane and affecting the discharge of nutrients, ultimately leading to a decrease in fat digestibility. The combination of polyphenols with digestive enzymes in animals and enzymes secreted by microorganisms might also result in a decrease in fat digestibility.

### 2.5. Effects of Proanthocyanidins with Different Degrees of Polymerization on Ca and Zn Digestibility

The apparent digestibility of calcium and zinc was lower in the experimental groups than the control group (*p* < 0.05; Table 3). These values were lower in the high-polymer group than the low-polymer group, but the difference was not significant (*p* > 0.05). The decrease in apparent digestibility of calcium and zinc in the experimental groups might have been due to the formation of chelates of polyphenols and metal ions that are poorly digested and absorbed by the body [17]. In addition, polyphenols may damage the intestinal mucosa by binding to proteins on the mucosa of the small intestine, thus reducing the bioavailability of mineral elements. Under our experimental conditions, both low- and high-polymer proanthocyanidins reduced the digestibility of Ca and Zn in mice, with no significant differences between these values.

## 3. Discussion

Energy and protein are the core factors affecting the metabolism and growth performance of animals. A deficiency or imbalance in dietary protein and energy can affect lean tissue deposition and protein turnover, resulting in low weight gain and feed conversion rates [18].

Proteins are a basic component of living cells and tissues that play key roles in numerous life activities. Protein digestion and absorption disorders affect human growth and development. In the current study, the apparent digestibility of proteins in the high-polymer group was significantly reduced, which was likely detrimental to the growth of mice. The apparent digestibility of proteins was significantly lower in mice in the high- vs. low-polymer group. Perhaps high-molecular-weight proanthocyanidins substantially alter the conformation of proteins, thereby affecting their digestibility and absorption. 

The combination of polyphenols and amino acids can affect the quantity and proportion of certain essential amino acids, and the reduced nutritional value of the protein will inevitably affect normal growth and development [19]. Indeed, the reaction of polyphenols with soy proteins blocks the absorption of lysine, tryptophan, and cysteine, thereby reducing the bioavailability of essential amino acids, as revealed by Rawel et al. [20]. Moreover, since the enzyme protease is a protein, the inhibition of protease activity also affects protein digestion. 

In the current study, high-polymer proanthocyanidins had a strong effect on proteases and to some extent also affected the biodegradation of proteins. Because the weak binding forces within these molecules have little effect on the conformation of proteins and proteases, they do not substantially alter the biological activity or physiological functions of proteins. Therefore, oligomeric proanthocyanidins are less likely to interfere with the absorption and utilization of proteins by the organism than are high-polymer proanthocyanidins. The diverse structures of proanthocyanidins make their interactions with proteins stereospecific. The degree of binding between these molecules is not only affected by the degree of polymerization of proanthocyanidins, but also by factors such as the molecular shape, hydrodynamic radius, phenolic hydroxyl position, degree of acylation, and steric hindrance. Although the number of active sites on polyphenols available for protein binding increases proportionally with the number of structural units of flavonols, this number is not proportional to the amount of precipitate obtained. In vitro, the affinity of catechin for proline is stronger than that of epicatechin. According to De et al. [21], the affinity of proanthocyanidin B3 and B4 (C4-C8) for proline is greater than that of their analogs B6 and B8 (C4-C6), respectively, whereas the degrees of specific binding of proanthocyanidin C1 (trimer) to proline, proanthocyanidins B2 (dimer), and epicatechin (monomer) are similar. A similar experiment using proanthocyanidins with different degrees of polymerization could be performed in the future.

The combination of polyphenols and the active sites of digestive enzymes (the phenolic hydroxyl groups present in polyphenols bind to the peptidyl groups of the enzymes (-NH-CO-, amino-NH_2_-, and carboxyl-COOH) via hydrogen bonds) alters the molecular structure of the enzyme, for instance by reducing the number of free amino acids and altering the structure of the amino acid side chain, resulting in a decrease or loss of biological activity, and thereby affecting the digestion and absorption of carbohydrates. Goncalves et al. [13] confirmed the wider interaction between α-amylase and high-polymer proanthocyanidins through fluorescence quenching, dynamic light scattering analysis, and turbidimetry. In the current study, the rate of inhibition of α-amylase activity in the high polymer group was close to 50%, which would reduce the biodegradation of carbohydrates, and thus limit growth and development. As shown in the above table, the affinity between polyphenols and various enzymes differed. The rate of α-amylase inhibition was greater than that of other digestive enzymes, which may be related to the stereostructure of the enzyme, the molecular weight, and/or the number of specific amino acids on the surface of the enzyme molecule. 

Compared to other digestive enzymes, amylase was more strongly affected by proanthocyanidin solution, likely because many amino acid residues on its surface bind to proanthocyanidins. The digestion and absorption of carbohydrates are also closely related to the presence of a glucose transporter. Johnston et al. [22] found that proanthocyanidins can inhibit the transport of glucose by inhibiting the activity of glucose transporter II, thereby reducing the rate of glucose release and absorption in the small intestine. 

Polyphenols can alter the emulsifying properties of fat by binding to the lipid layer or the hydrophilic head of lecithin outside the emulsion droplets [23]. In the current study, fat digestibility in mice was lower in the high-polymer group than the low-polymer group. Perhaps the high-molecular-weight polyphenols function as connectors between the complexes, thereby increasing the droplet size and inhibiting the digestion and absorption of fat. According to Haslam [24], the mechanism of polyphenol–lipid complex formation is similar to the polyphenol–protein binding mechanism, which is based on hydrogen bonds and hydrophobic bonds. The high-polymer group of mice had lower fat digestibility, possibly due to the stronger binding between the high-polymer proanthocyanidins and lipids, which would limit the biodegradation of pancreatic lipase. Compared to pancreatic amylase and trypsin activity, the inhibitory effect of proanthocyanidins on pancreatic lipase activity is generally small, perhaps because this enzyme has fewer amino acid residues that bind to proanthocyanidins due to its spatial configuration and smaller molecular weight. Recent studies on the effects of polyphenols on pancreatic lipase activity have yielded mixed results. Sugiyama et al. performed in vitro simulated digestion experiments that showed that catechin and epicatechin have no inhibitory effect on pancreatic lipase activity. As the degree of polymerization (dimer–pentamer) increased, the inhibitory effects of proanthocyanidins on pancreatic lipase activity increased. The inhibition of pancreatic lipase activity by proanthocyanidins above the polymer is not much different [25]. Griffiths et al. [26] demonstrated that polyphenols inhibit murine α-amylase and trypsin activity and promote pancreatic lipase activity. Longstaff et al. [27] found that polyphenols inhibited α-amylase, trypsin, and pancreatic lipase activity in chickens, but when the protein content in the feed increased to a certain level, polyphenols increased pancreatic lipase activity, perhaps due to the stronger binding of protein molecules to polyphenols, which weakened the effects of polyphenols on lipase. The results of experiments investigating the effects of polyphenols on pancreatic lipase activity may be affected by factors such as experimental methods, experimental subjects, and polyphenol types.

## 4. Materials and Methods 

### 4.1. Separation and Measurement of Proanthocyanidins with Different Degrees of Polymerization 

Proanthocyanidins were isolated from grape (*Vitis vinifera*) seeds by the methanol-chloroform solvent two-phase precipitation method [28]. Proanthocyanidin levels and quality were determined by the vanillin-glacial acetic acid method, and the average degree of polymerization of the samples was calculated by combining the average molecular weights of the compounds [29]. Animal feeding experiments were performed using fragments with degrees of polymerization of 2–4 units and >10 units for the low- and high-polymer group, respectively.

### 4.2. Feed Management and Sample Collection

Twenty-four adult male-specific pathogen-free Wistar rats (Chinese Academy of Medical Sciences Animal Breeding Center, Beijing, China) were used in the experiments. Animal experiments were performed according to ethical standard issued by the National Institutes of Health Guide for the Care and Use of Laboratory Animals and approved by Institutional Animal Care and Use Committee.

The conditions of the animal breeding room were 20–25 °C, 55–60% humidity, and a 12 h:12 h light/dark cycle. The mice were free to eat and drink for one week. After 7 days of laboratory conditioning, the mice were randomly divided into three groups of eight animals per group. The mice were fed intragastrically once per day at 3 pm for 8 weeks. The first group of mice was fed with distilled water as a blank control. The second group was fed with an oligomeric proanthocyanidin solution at a dose of 150 mg/kg. The third group was fed with a high-polymer proanthocyanidin solution at a dose of 150 mg/kg. The mice ate basal feed freely, and weight gain and feed consumption were recorded weekly. The mouse feces were collected and weighed for 6–8 weeks. After the experiment, all experimental mice were sacrificed in the morning on an empty stomach, the rats were euthanized by lethal intraperitoneal injection of pentobarbital (150 mg/kg; <200 mg/mL) and quickly dissected. Subsequently, the contents of the small intestine and stomach were collected, and the pancreas was collected, weighted and homogenized in 1: 9 (*w*/*v*) physiological saline for 30 s by using the FJ-200 type high-speed tissue homogenizer, then centrifuged at 4 °C for 10 min and placed in liquid nitrogen, and transferred to a −80 °C freezer for further assays [30].

### 4.3. Determination of Food Utility Rate

Food utility rate was calculated as follows:

Food utility rate (%) = weight gain (g)/feed intake (g)(1)

### 4.4. Determination of Enzyme Activity

The activities of alpha-amylase, pancreatic lipase, trypsin, and pepsin were determined using commercial kits purchased from Nanjing Jiancheng Institute of Bioengineering (Nanjing, China) according to the manufacturer’s instructions. 

Digestive enzyme activity was calculated as follows:

Digestive enzyme activity = (digestive enzyme activity in the control group − digestive enzyme activity in the experimental group)/digestive enzyme activity in the control group(2)

### 4.5. Determination of Protein Digestibility

The content of crude protein in feed and feces was determined by the Kjeldahl method. Each feed or feces sample was weighed, sequentially combined with copper sulfate, potassium sulfate, and concentrated sulfuric acid, gently shaken, and heated with a small flame in an electric furnace. After the contents were completely carbonized and foam generation had stopped, the flame intensity increased. After the liquid turned blue-green, the sample was heated for a bit longer, removed from the furnace, and cooled to a constant volume. The reaction was carried out using NaOH solution in a Auto-Kjeldahl Apparatus (KDY–9820, China). NH_3_ was released by distillation and collected in H_3_BO_3_ solution. Titration was carried out using a known concentration of a sulfuric acid standard solution, and the nitrogen content was calculated based on the consumption of H_2_SO_4_. The nitrogen content was multiplied by 6.25, and the protein content was calculated as follows:

Apparent protein digestibility (%) = (intake of nitrogen − fecal nitrogen)/intake of nitrogen × 100(3)

### 4.6. Determination of Fat Digestibility 

Soxhlet extraction techniques were used to determine the crude fat content of feed and feces. A 2 g sample of ground feed or feces was transferred to a tube of filter paper, which was placed into a Soxhlet extraction tube. Anhydrous ether was added to the upper end of the extraction tube, and the sample was heated in a water bath for reflux extraction until the extraction was complete. After removing the fat receiving bottle, the remaining ether in the water bath was evaporated, and the fat receiving bottle was placed in a desiccator and dried. The operation was repeated until a constant weight was achieved, and the increase in weight of the receiving bottle was used to calculate the crude fat content in the sample.

Apparent digestibility of fat (%) = (intake of fat − fat in feces)/intake of fat × 100(4)

### 4.7. Determination of Minerals Digestibility 

The calcium and zinc contents in animal feed and feces were determined by atomic absorption spectrometry. After placing 1 g of ground feed or feces into a crucible, the samples were placed on an electric heating plate for low-temperature carbonization. The sample was completely carbonized, and the crucible was transferred to a muffle furnace at 550 °C for high-temperature ashing. After adding HCl to dissolve all inorganic elements in the ash, the sample was diluted to a constant volume. The sample was introduced into the air-acetylene flame of the atomic absorption spectrophotometer (Perkin-Elmer Corp., Shelton, CT, USA), and the Ca and Zn contents were quantitatively determined using the standard curve method. The apparent digestibility of calcium and zinc was calculated according to the following formulas:

Apparent absorption rate of calcium and zinc (%) = (intake of calcium and zinc − amount of calcium and zinc in the feces)/intake of calcium and zinc × 100(5)

Intake of calcium and zinc (mg/day) = contents of calcium and zinc in feed/% amount of feed consumption (mg/day)(6)

Amount of fecal calcium and zinc (mg/day) = contents of calcium and zinc in feces/% × amount of fecal output (mg/day)(7)

### 4.8. Statistical Analysis

The experimental data for each treatment group were expressed as the average ± standard deviation. The Duncan new repolarization difference method to analyze differences amongst multiple samples. Correlation analysis was performed using SPSS 17.0 software (SPSS Inc., Chicago, IL, USA). Differences between samples were considered to be significant at *p < 0.05*.

## 5. Conclusions

Our 56-day intragastric experiment suggested that treatment with high-polymer proanthocyanidins had a significant effect on digestive enzyme activity and the digestion and absorption of nutrients in mice. The anti-nutritional properties of polyphenols depend on their content [31]. The current study confirmed that the high degree of polymerization of proanthocyanidins is a major reason for their antinutritional properties. Therefore, during the process of food production, the anti-nutritional properties of polyphenols could be minimized by reducing the degree of polymerization of proanthocyanidins. However, in this study, we only explored the effects of proanthocyanidins with different degrees of polymerization on nutrient digestion and digestive enzyme activities in vivo from a macroscopic perspective. The specific reaction patterns were not studied in depth. We plan to conduct a follow-up investigation on this metabolic mechanism at the cellular or molecular level, by analyzing the amino acid sequences, secondary structures, and conformational changes of the complexed proteins. Such studies should provide scientific guidelines for including proanthocyanidin use in the food, chemical, pharmaceutical, and other industries, thereby improving the comprehensive utilization rate of these beneficial compounds.

## Figures and Tables

**Table 1:** Effects of procyanidins with different degrees of polymerization on body weight gain, food consumption, and the feed conversion ratio in mice.

Group	Initial Weight (g)	Final Weight (g)	Weight Increase (g)	Total Feed Intake (g)	Food Utility Rate (%)
Control	28.92 ± 2.04 ^a^	40.81 ± 2.45 ^a^	11.89 ± 1.45 ^a^	276.26 ± 10.43 ^a^	4.30 ± 0.45 ^a^
Low polymer	29.01 ± 1.78 ^a^	39.19 ± 2.78 ^a^	10.18 ± 0.98 ^a^	244.26 ± 7.45 ^b^	4.17 ± 0.39 ^a^
Highpolymer	29.41 ± 1.56 ^a^	37.94 ± 3.12 ^b^	8.53 ± 1.01 ^b^	242.22 ± 9.09 ^b^	3.52 ± 0.52 ^b^

^a, b^ Differentsuperscripts in the same line indicate significant differences (*p* < 0.05).

**Table 2:** Effects of procyanidins with different degrees of polymerization on digestive enzyme activity in mice ^1^.

Relative Enzyme Activity	Control Group	Low-Polymer Group	Rate of Inhibition of Digestive Enzyme Activity in the Low-Polymer Group	High-Polymer Group	Degree of Digestive Enzyme Activity Inhibition in the High-Polymer Group
α-amylase activity in small intestine	0.34 ± 0.05 ^a^	0.27 ± 0.06 ^b^	21%	0.20 ± 0.03 ^c^	41%
α-amylase activity in the pancreas	0.47 ± 0.05 ^a^	0.35 ± 0.04 ^b^	26%	0.26 ± 0.04 ^c^	45%
Pancrelipase activity in the small intestine	2.21 ± 0.08 ^a^	2.05 ± 0.2 ^a^	7%	1.99 ± 0.16 ^a^	10%
Pancrelipase activity in the pancreas	3.28 ± 0.21 ^a^	2.95 ± 0.25 ^a^	10%	2.84 ± 0.39 ^a^	13%

^a, b^ Different superscripts in the same line indicate significant differences (*p* < 0.05); digestive enzyme activity unit (U mg prot^−1^). ^1^ The rate of inhibition by proanthocyanidins with different degrees of polymerization on digestive enzyme activity = (Digestive enzyme activity of control group—Digestive enzyme activity of experimental group)/Digestive enzyme activity of control group.

**Table 3:** Effects of procyanidins with different degrees of polymerization on nutrient absorption in mice.

Apparent Digestibility	Control Group	Low-Polymer Group	High-Polymer Group
Apparent digestibility of protein (%)	86.33 ± 0.67 ^a^	84.89 ± 1.36 ^a^	78.53 ± 1.91 ^b^
Apparent digestibility of fat (%)	87.41 ± 0.98 ^a^	86.08 ± 0.51 ^a^	82.65 ± 1.91 ^b^
Apparent digestibility of calcium (%)	33.01 ± 2.12 ^a^	29.06 ± 3.08 ^b^	27.97 ± 1.85 ^b^
Apparent digestibility of zinc (%)	28.86 ± 0.80 ^a^	24.45 ± 1.47 ^b^	23.38 ± 1.50 ^b^

^a, b^ Different superscripts in the same line indicate significant differences (*p*< 0.05).
